# Radiographic Anatomy of the Lateral Ankle Ligament Complex: A Cadaveric Study

**DOI:** 10.1177/10711007231213355

**Published:** 2023-11-23

**Authors:** Jordan B. Robbins, Shepheard A. Stahel, Randal P. Morris, Daniel C. Jupiter, Jie Chen, Vinod K. Panchbhavi

**Affiliations:** 1 John Sealy School of Medicine, The University of Texas Medical Branch, Galveston, TX, USA; 2 Department of Orthopaedic Surgery and Rehabilitation, The University of Texas Medical Branch, Galveston, TX, USA; 3 Department of Biostatistics and Data Science, The University of Texas Medical Branch, Galveston, TX, USA

**Keywords:** anatomic landmarks, ankle instability, ankle lateral ligaments, anterior talofibular ligament, calcaneofibular ligament, fluoroscopy, minimally invasive surgery

## Abstract

**Background:**

When lateral ankle sprains progress into chronic lateral ankle instability (CLAI), restoring precise anatomic relationships of the lateral ankle ligament complex (LALC) surgically is complex. This study quantifies the radiographic relationships between the anterior talofibular ligament (ATFL), calcaneofibular ligament (CFL), and prominent osseous landmarks visible under fluoroscopy to assist in perioperative practices for minimally invasive surgery for CLAI.

**Methods:**

Ten fresh frozen ankle specimens were dissected to expose the LALC and prepared by threading a radiopaque filament through the ligamentous footprints of the ATFL and CFL. Fluoroscopic images were digitally analyzed to define dimensional characteristics of the ATFL and CFL. Directional measurements of the ligamentous footprints relative to the lateral process of the talus and the apex of the posterior facet of the calcaneus were calculated.

**Results:**

Dimensional measurements of the ATFL were a mean length of 9.3 mm, fibular footprint of 9.4 mm, and talar footprint of 9.1 mm. Dimensional measurements of the CFL were a mean length of 19.4 mm, fibular footprint of 8.2 mm, and calcaneal footprint of 7.3 mm. From the radiographic apparent tip of the lateral process of the talus, the fibular attachment of the ATFL was found 13.3 mm superior and 4.4 mm posterior, whereas the talar attachment was found 11.5 mm superior and 4.8 mm anterior. From the radiographic apparent posterior apex of the posterior facet of the calcaneus, the fibular attachment of the CFL was found 0.2 mm inferior and 6.8 mm anterior, whereas the calcaneal attachment was found 14.3 mm inferior and 5.9 mm posterior.

**Conclusion:**

The ATFL and CFL were radiographically analyzed using radiopaque filaments to outline the ligamentous footprints in their native locations. These ligaments were also localized with reference to 2 prominent osseous landmarks. These findings may assist in perioperative practices for keyhole incision placement and arthroscopic guidance. Perfect lateral ankle joint imaging with talar domes superimposed is required to be able to do this.

**Clinical Relevance:**

Radiographic evaluation of the ATFL and CFL with reference to prominent osseous landmarks identified under fluoroscopy may assist in perioperative practices for minimally invasive surgery to address CLAI for keyhole incision placement and arthroscopic guidance.

## Graphical abstract

**Graphical abstract:**
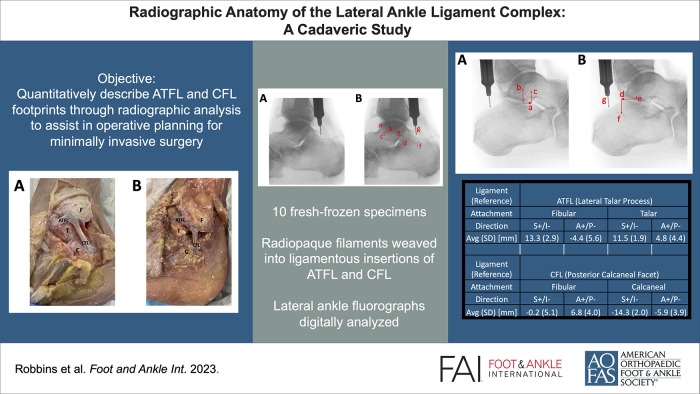

## Introduction

Acute ankle sprains are some of the most common orthopaedic injuries, occurring across a broad demographic range at a rate of nearly 2 million annually in the United States.^1,14,15,30,34^ More than 75% of ankle sprains include the lateral ankle ligament complex (LALC), which is composed of the anterior talofibular ligament (ATFL), the calcaneofibular ligament (CFL), and the posterior talofibular ligament (PTFL). Approximately 70% to 85% of these lateral ankle sprains involve the ATFL and more than half involve the CFL.^7,14,30^ The PTFL is the strongest ligament of the 3 and is rarely injured.^7^ High recurrence of ankle sprains leads to chronic lateral ankle instability (CLAI), defined as laxity and mechanical instability, of the ankle that interferes with activity. CLAI may develop from repeated sprains or even from a single injury.^1,10,32^ In these instances, surgery is indicated to restore function and stability when nonoperative measures have failed.^4,12,22^

Surgery to address CLAI can be categorized into 3 groups: anatomic repair, anatomic reconstruction, and nonanatomic reconstruction. Nonanatomic reconstruction uses tendon grafts at locations other than that of the native anatomy to mimic the function of the LALC. This approach is not recommended because of significantly higher rates of complications, such as iatrogenic nerve injury, subsequent sprains, long-term stiffness, and degeneration.^4,5,12,22^ Conversely, anatomic interventions have proven more effective in restoring function for patients with CLAI and are often the preferred surgical option.^4,5,12,22,29,33,36^ The gold standard for anatomic repairs is the modified Brostrom-Gould procedure, which uses the inferior extensor retinaculum to strengthen the repair of the ATFL.^4,9,11,12,22,26,29,36^ Anatomic repair may be contraindicated in patients with insufficient native tissue, a high body mass index, high-level athletes, or a previously failed anatomic repair.^4,12,21,22,29^ In these cases, an anatomic reconstruction with an autograft or allograft may be preferred.

Although there is some debate regarding the choice between anatomic repair and reconstruction regarding better clinical outcomes, the consensus from the literature stresses the importance of restoring the precise anatomy of the ATFL and CFL.^21,26,29^ Although many studies have defined the quantitative parameters of the LALC and the surrounding structures, few have sought to incorporate radiographic analysis for the precise anatomy of the ligaments.^2,3,6,8,13,16-20,23-25,27,28,31,35^ These radiographic data could serve to augment preoperative preparation and intraoperative guidance, especially in the context of percutaneous and arthroscopic approaches.

The purpose of our study is to expand on previous efforts to radiographically evaluate the LALC by defining the natural anatomic location of the ATFL and CFL on standard radiographs in order to aid in perioperative practices of minimally invasive surgery. We hypothesize that the ATFL and CFL footprints can be precisely illustrated on radiographs in relation to prominent osseous landmarks in a reproducible manner.

## Methods

### Specimen Preparation

Sixteen fresh frozen cadaveric specimens from 9 total donors with no identifiable history of ankle osteoarthritis, osteoporosis, ankle fractures, or previous ankle surgeries were initially dissected by 1 author (J.R.) under direct supervision and instruction from the senior author (V.P.). Dissections were carefully carried out to reflect skin, connective tissue, muscles, tendons, and neurovascular structures to expose the ATFL and CFL in their native locations at the lateral ankle joint. After gross dissection, 6 specimens with apparent anatomical defects were excluded. These defects included 3 specimens with a disorganized confluence of the fibular attachment site of the ATFL and CFL, 2 specimens with a missing ATFL, and 1 specimen with a missing CFL. These defects were likely the result of fibrous scar tissue formation following significant prior injury. The remaining 10 specimens were from 7 total donors (4 male, 3 female). The demographics of the donors, listed as mean (SD), were as follows: age 66.4 (12.2) years, height 170.9 (6.9) cm, weight 81.2 (30.3) kg, and body mass index 27.7 (10.1). No specimens exhibited gross deformities in midfoot arch architecture.

**Figure 1.**
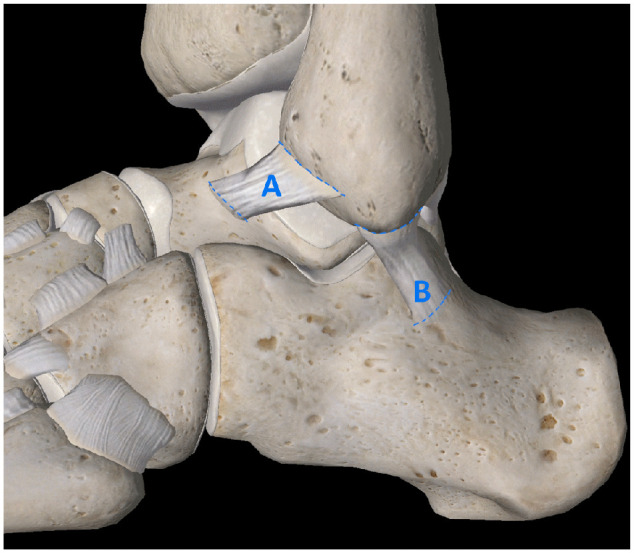
Representative anatomical diagram of specimen preparation demonstrating RAY-TEC filaments (dashed lines) weaved into footprints of (A) ATFL and (B) CFL. Image courtesy of Complete Anatomy (3D4Medical).

**Figure 2.**
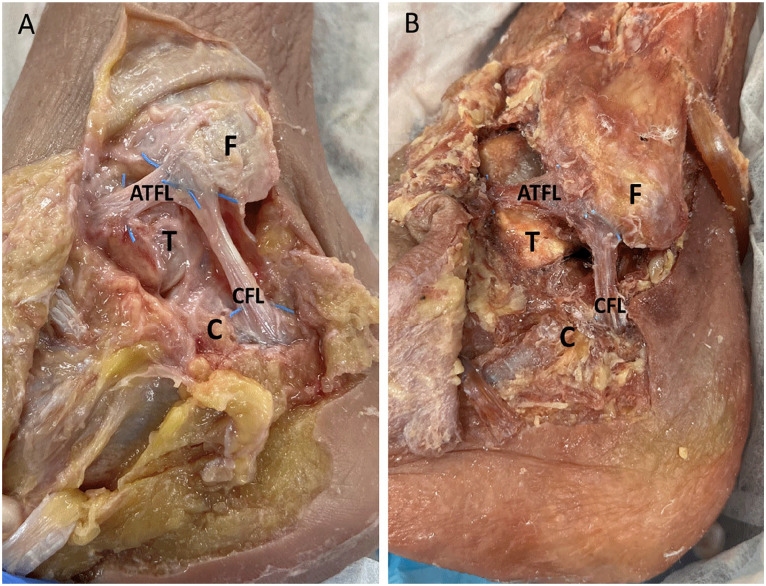
Gross anatomic dissection images. (A) Untrimmed RAY-TEC filaments weaved into the bony attachment sites of the ATFL and CFL footprints. (B) Trimmed RAY-TEC filaments ready for radiographic imaging. (F) Fibula. (T) Talus. (C) Calcaneus.

The 10 specimens used in this study were prepared by using a free needle to weave a radiopaque filament obtained from a RAY-TEC lap sponge (Johnson & Johnson, New Brunswick, NJ) across the footprints of the bony attachment sites of the ATFL and CFL (Figure 1). Ligaments were left unaltered in their native locations, and loose ends of the radiopaque markers were trimmed flush to the borders of the ATFL and CFL before imaging (Figure 2).

### Radiographs and Image Analysis

Using miniature C-arm fluoroscopy, lateral radiographic images of each ankle held in neutral alignment were captured. True laterals were defined as the lateral malleolus superimposed over the medial malleolus with the talar domes superimposed, showing a confluent superior articular surface. Each radiographic image was captured with a length-standard depth gauge set at 10 mm held perpendicular to the C-arm and level with the lateral ankle ligaments for measurement calibration.

**Figure 3.**
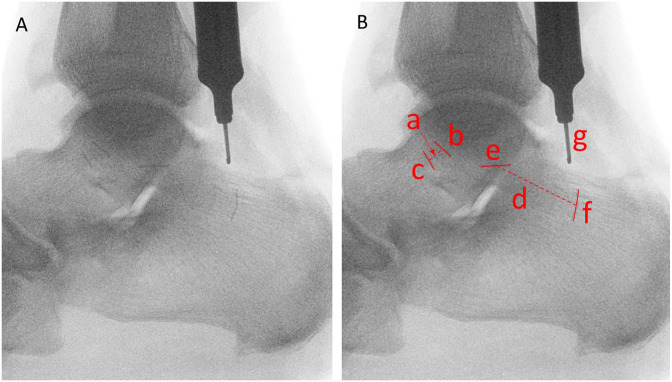
Dimensional measurements for ATFL and CFL. (A) Original radiograph highlighting RAY-TEC wire at the ligamentous footprints on the fibula, talus, and calcaneus. (B) Demonstration of image analysis performed to define ligament dimensions: (a) ATFL length, (b) ATFL fibular footprint, (c) ATFL talar footprint, (d) CFL length, (e) CFL fibular footprint, (f) CFL calcaneal footprint, (g) length-standard depth gauge at 10 mm.

Lateral ankle radiographs were digitally analyzed using ImageJ (National Institutes of Health) by 1 author (S.S.) under direct supervision and instruction from the senior author (V.P.). Using the 10-mm-depth gauge as calibration, each image was analyzed to define the characteristics of the ATFL and CFL ligaments at their respective fibular, talar, and calcaneal attachment sites as highlighted by the radiopaque markers. Dimensional measurements of the ATFL and CFL footprints were obtained by measuring the 1-dimensional length of each radiopaque marker, and the length of each ligament was calculated by averaging the straight-line span measurements taken from each of the 2 poles of each ligament’s respective radiopaque markers (Figure 3). Each respective length measurement was obtained 4 times with image analysis software and averaged to strengthen reliability.

**Figure 4.**
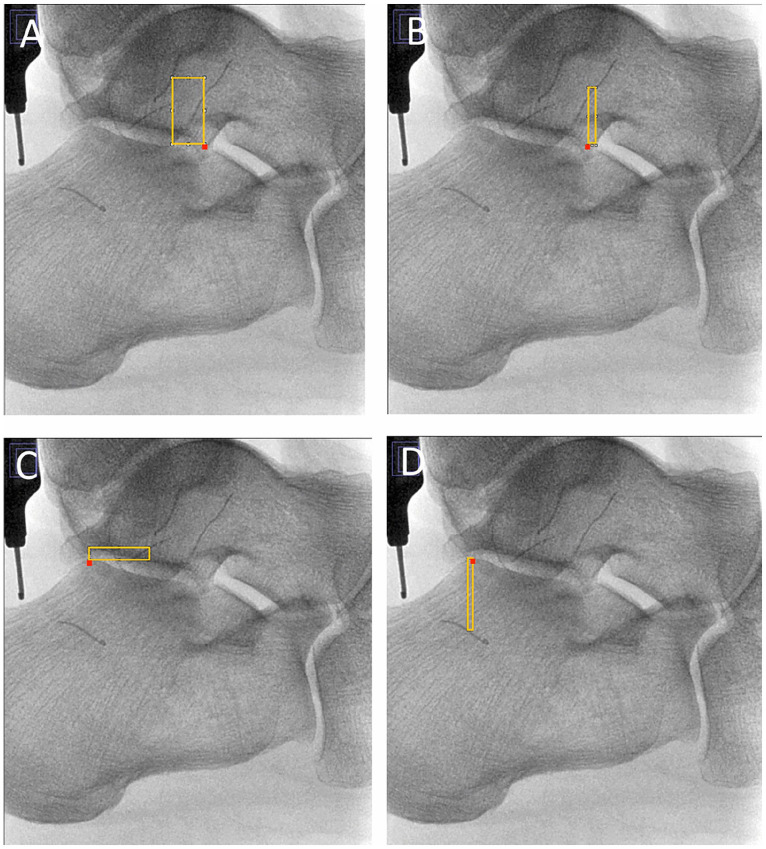
Directional measurement vectors used to find the relative distance from the lateral talar process to the (A) fibular and (B) talar attachments of the ATFL and from the apex of the posterior facet of the calcaneus to the fibular (C) and calcaneal (D) attachments of the CFL. The lateral process of the talus and the apex of the posterior facet of the calcaneus are illustrated with red boxes. [See online article for color figure.]

**Figure 5.**
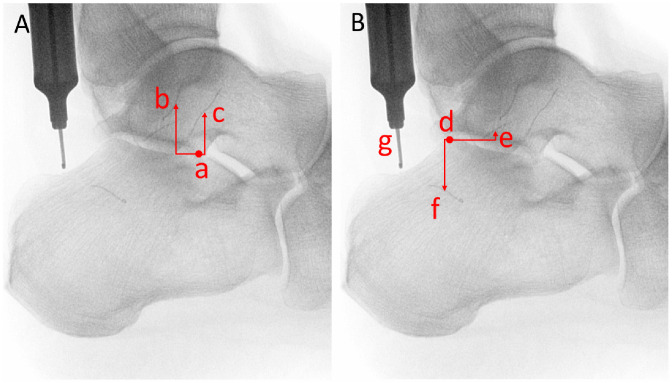
Directional measurements for ATFL and CFL in reference to respective bony prominences. (A) Directional measurements for fibular (b) and talar (c) footprints of the ATFL in reference to the lateral talar process (a). (B) Directional measurements for fibular (e) and calcaneal (f) footprints of the CFL in reference to the posterior calcaneal facet (d), and length-standard depth gauge at 10 mm (g).

Directional measurements were obtained for each ligament with reference to 2 different radiographic landmarks, as defined by the senior author (V.P.). The lateral process of the talus identified by the apex seen on true lateral radiographs was designated for the landmark of the ATFL. The apex of the posterior facet of the calcaneus seen on true lateral radiographs was designated for the landmark of the CFL. Figures 4 and 5 each highlight the exact designation of both radiographic landmarks. After establishing orthogonal axes, the rectangle function in ImageJ was used to create a reliable measurement template in the vectors of interest (Figure 4). Each template was started at the midpoint of the ATFL or CFL and completed at the lateral process of the talus or the apex of the posterior facet of the calcaneus, respectively. Measurements were then obtained 4 times from the template and averaged to strengthen reliability (Figure 5). Superior and anterior directions were defined as positive, whereas inferior and posterior directions were defined as negative.

### Statistical Analysis

Descriptive statistics were calculated for dimensional and directional measurements in Microsoft Excel, version 16.69.1 (Microsoft Corp). Measurement lengths were reported in millimeters as mean (SD).

## Results

Dimensional measurements for the ATFL and CFL, as demonstrated in Figure 3, are listed in Table 1. The length of the ATFL was 9.3 (4.5) mm, with the fibular footprint measuring 9.4 (2.7) mm and the talar footprint as 9.1 (3.2) mm. The length of the CFL was 19.4 (3.7) mm, with the fibular footprint measuring 8.2 (2.4) mm and the calcaneal footprint 7.3 (1.8) mm. When stratified by sex, male specimens had a statistically longer CFL than female specimens, with lengths measuring 21.5 (3.2) mm and 16.2 (1.6) mm, respectively. No further statistical differences were identified between male and female specimens.

**Table 1.** Descriptive Statistics for ATFL and CFL Dimensions as Measured Through Image Analysis Software From Radiographs (N = 10).^a^

Ligament	Measurement, mm, mean (SD)
ATFL	
Length	9.3 (4.5)
Fibular footprint	9.4 (2.7)
Talar footprint	9.1 (3.2)
CFL	
Length	19.4 (3.7)
Fibular footprint	8.2 (2.4)
Calcaneal footprint	7.3 (1.8)

Abbreviations: ATFL, anterior talofibular ligament; CFL, calcaneofibular ligament.

a Radiopaque filaments defined ligamentous footprints and were measured directly. Ligament lengths were determined indirectly by averaging straight-line span measurements taken from each of the 2 poles of each ligament’s respective radiopaque markers.

Directional measurements for the ATFL and CFL with respect to their bony reference points, as demonstrated in Figure 5, are listed in Table 2. From the lateral talar process, the fibular attachment of the ATFL was 13.3 (2.9) mm superior and 4.4 (5.6) mm posterior, and the talar attachment of the ATFL was 11.5 (1.9) mm superior and 4.8 (4.4) mm anterior. From the apex of the posterior facet of the calcaneus, the fibular attachment of the CFL was 0.2 (5.1) mm inferior and 6.8 (4.0) mm anterior, and the calcaneal attachment of the CFL was 14.3 (2.0) mm inferior and 5.9 (3.9) mm posterior. When stratified by sex, no statistical differences were identified between male and female specimens for directional measurements in either ligament.

**Table 2.** Descriptive statistics for directional measurements of ATFL and CFL in reference to respective bony prominences (N = 10). Measurements listed in mm.

Ligament (reference)	Directional Measurement, mm, Mean (SD)	
ATFL (lateral talar process)		
	S+/I–	A+/P–
Fibular	13.3 (2.9)	−4.4 (5.6)
Talar	11.5 (1.9)	4.8 (4.4)
CFL (posterior calcaneal facet)		
Fibular	−0.2 (5.1)	6.8 (4.0)
Calcaneal	−14.3 (2.0)	−5.9 (3.9)

Abbreviations: A+/P–, anterior (+) / posterior (–); ATFL, anterior talofibular ligament; CFL, calcaneofibular ligament; S+/I–, superior (+) / inferior (–).

## Discussion

Regarding surgical management of CLAI, the restoration of the precise anatomy of the ATFL and CFL has proven paramount for favorable clinical outcomes.^4,5,12,21,22,26,29,33,36^ Although several previous studies have addressed the quantitative characteristics of the ATFL and CFL, most have been conducted for the purpose of traditional open surgery with reference to osseous landmarks detected by palpation or following gross dissection.^2,6,13,23-25,27,31^ Our study expands on these efforts, especially within the context of minimally invasive surgery through keyhole incisions and arthroscopy. We found that the footprints of the ATFL and CFL can be reliably identified under fluoroscopic guidance with reference to 2 prominent bony landmarks: the lateral talar process and the apex of the posterior facet of the calcaneus.

By weaving a radiopaque filament across the ATFL and CFL footprints, we were able to quantitatively evaluate the entire ligaments radiographically through image analysis. The measured dimensions of the ATFL and CFL in our study closely match those in the published literature. Taser et al^31^ conducted a cadaveric study over the LALC in a traditional open fashion to measure distances and angles from bony landmarks and to qualitatively evaluate the ligaments using radiopaque lead paint. They found the ATFL fibular footprint and talar footprint lengths to be 10.8 (1.6) mm and 11.0 (2.4) mm, respectively. Our study similarly found ATFL fibular footprint and talar footprint lengths to be 9.4 (2.7) mm and 9.1 (3.2) mm. Likewise, our CFL measurements for the fibular footprint and calcaneal footprint, 8.2 (2.4) mm and 7.3 (1.8) mm, closely approximate measurements from Taser et al, 7.2 (2.2) mm and 9.7 (1.7) mm.^31^

In another recent study, Haytmanek et al^13^ used radiopaque beads implanted into the center of each footprint to radiographically evaluate the LALC. They found the lengths of the ATFL and CFL to be 9.4 (2.4) mm and 28.1 (4.8) mm. By measuring the spans between each respective footprint, our study found the average ATFL and CFL lengths to be 9.3 (4.5) mm and 19.4 (3.7) mm. These results also closely follow those published in a systematic review regarding the anatomy of the LALC by Matsui et al,^24^ which found the lengths of the ATFL and CFL to be 12.0 to 24.8 mm and 18.5 to 35.8 mm.

By outlining the ligamentous footprints with radiopaque markers, we were also able to determine directional measurements from prominent osseous landmarks identified under fluoroscopy. Specifically, we chose the lateral talar process and the apex of the posterior facet of the calcaneus for the ATFL and CFL, respectively. These reference points were chosen to expand on previous landmarks published in the literature and to provide reliable alternatives, especially for the calcaneal attachment of the CFL. Previous studies have commonly found landmarks on the lateral malleolus and the talus to be reliable references for localizing the ATFL insertions.^6,13,23,24,27^ Authors of other studies have investigated the peroneal tubercle and the tuberculum ligamenti calcaneofibularis as references for CFL insertions, but these landmarks have proven less reliable without traditional open visualization.^6,13,23,27^

With reference to the lateral talar process, we found the talar attachment of the ATFL to be 11.5 (1.9) mm superior and 4.8 (4.4) mm anterior. This closely follows results published by Haytmanek et al^13^ as 12.0 mm superior and 6.0 mm anterior. Likewise, Clanton et al^6^ conducted a traditional open cadaveric study on the LALC and found the talar attachment of the ATFL to be 17.8 mm superior from the lateral talar process measured along the anterior border of the lateral talar articular facet. To our knowledge, no prior studies have investigated the directional relationships of the fibular insertion of the ATFL in reference to the lateral talar process. We found this insertion for the fibular footprint to be 13.3 (2.9) mm superior and 4.4 (5.6) mm posterior from the lateral talar process.

Posed as a novel and more reliable alternative to previously published landmarks, the apex of the posterior facet of the calcaneus was chosen for the osseous reference point of CFL insertions in our study. We found the fibular footprint of the CFL to originate 0.2 (5.1) mm inferior and 6.8 (4.0) mm anterior to this landmark, whereas the calcaneal footprint originated 14.3 (2.0) mm inferior and 5.9 (3.9) mm posterior. Although this landmark has yet to be investigated in the literature, Matsui et al^23^ found the distal CFL insertion to be an absolute distance of 17.2 (range, 14.4-21.0) mm from the midpoint of the lateral border of the posterior facet of the subtalar joint in their traditional open cadaveric study. Likewise, the systematic review from Matsui et al^24^ found the inferior distance of the distal CFL insertion to be 12.1 to 13.2 mm from the midpoint of the subtalar joint.

Our study offers several advantages to anatomical studies of the LALC. The use of fresh frozen specimens allowed for optimal preservation of anatomy compared to embalmed specimens. The implementation of radiopaque filaments highlighting the ligamentous footprints of the ATFL and CFL was a novel approach to radiographic analysis of the LALC. This approach allowed us to analyze the complete ligamentous anatomy without disturbing the native anatomic locations during fluoroscopy. For dissections and measurements, each was performed by 1 individual under direct observation and guidance of the senior author in order to mitigate interrater variability. The benefits of the chosen osseous landmarks must also be mentioned. The authors of this study found the lateral talar process and the apex of the posterior facet of the calcaneus to be reliable landmarks appreciated on lateral fluoroscopic images both in terms of ease of identification and consistency in anatomic presentation in specimens. The critical nature of first obtaining a true lateral image before identifying these landmarks cannot be overstated, as rotationally skewed images may greatly influence the location of which these landmarks present on lateral radiographs. These landmarks provided directional measurements to ATFL and CFL insertions with suitable precision for the clinical context. The largest SD in our measurements was found to be 5.6 mm, whereas the minimum keyhole incision length used in minimally invasive surgery is often 10 mm or more. Given these parameters, we suggest that the footprints of the ATFL and CFL can be reliably accessed via primary keyhole incision in most patients.

Several limitations of our study must be acknowledged. The influence of aging and prior injury in the donors should be mentioned as several ankles were excluded following initial gross dissection due to missing, scarred, or confluent ligaments. The sample size of 10 viable specimens used in our study may not truly provide an accurate representation of the population, but this sample size is consistent with those of similar studies and presents reasonable results when contextualized with the literature. During fluoroscopic imaging of the isolated lower leg cadaveric specimens, there was no proxy for weightbearing, which made evaluation of midfoot deformities such as pes planus or cavus impossible. On image analysis, the reference of a depth gauge set to 10 mm presented an opportunity for systematic error and could be rectified by using a radiopaque ruler tape in future studies. Finally, the inherent differences in measuring straight-line radiographic lengths in 2 dimensions must be mentioned when comparing to other anatomic studies measuring contoured lengths in 3 dimensions. Yet, when compared to other published measurements, we think that our metrics determined from fluoroscopic images offered a high degree of clinical relevance to the operating room and did not require corroboration with 3-dimensional advanced imaging studies. Finally, we do not present any clinical evidence of efficacy, as this was a purely laboratory-based study and ideal conditions (no obvious bony or ligamentous pathology, complete control of specimen and image orientation, etc) were present.

Our study presents a unique radiographic evaluation of the ATFL and CFL with reference to 2 prominent osseous landmarks identified under fluoroscopy. These findings may assist in operative practices for surgery to address CLAI in keyhole incision placement and arthroscopic guidance. Further clinical efficacy studies are needed.

## Supplemental Material

sj-pdf-1-fai-10.1177_10711007231213355 – Supplemental material for Radiographic Anatomy of the Lateral Ankle Ligament Complex: A Cadaveric Study Click here for additional data file. Supplemental material, sj-pdf-1-fai-10.1177_10711007231213355 for Radiographic Anatomy of the Lateral Ankle Ligament Complex: A Cadaveric Study by Jordan B. Robbins, Shepheard A. Stahel, Randal P. Morris, Daniel C. Jupiter, Jie Chen and Vinod K. Panchbhavi in Foot & Ankle International
